# Wavelength-Flexible Thulium-Doped Fiber Laser Based on Digital Micromirror Array

**DOI:** 10.3390/mi11121036

**Published:** 2020-11-25

**Authors:** Xiao Chen, Dezheng Dai, Yi Zhang, Hongyuan Wu, Yunshu Gao, Genxiang Chen, Yiquan Wang

**Affiliations:** College of Science, Minzu University of China, Beijing 100081, China; DAIDEZHENG@126.com (D.D.); zy1173147374@163.com (Y.Z.); atomwhy@126.com (H.W.); gaoyunshu@126.com (Y.G.); gxchen_bjtu@163.com (G.C.); yqwang@muc.edu.cn (Y.W.)

**Keywords:** thulium-doped fiber laser, tunable laser, digital micromirror device

## Abstract

Wavelength-tunable thulium-doped fiber laser is demonstrated employing a digital micromirror device (DMD) in combination with a fixed grating. The diffraction property of four typical models of DMDs and its steering efficiency for the laser system are analyzed based on two-dimensional grating theory. By spatially modulating reflective patterns on a DMD, the stable, fast, and flexible tuning of lasing wavelength from 1930 nm to 2000 nm is achieved with wavelength tuning accuracy of 0.1 nm. The side-mode suppression ratio is larger than 50 dB around the 2 μm band with 3 dB linewidth less than 0.05 nm. The wavelength drift and power fluctuation are lower than 0.05 nm and 0.1 dB within 1 h at the room temperature, respectively.

## 1. Introduction

Space laser communication has the advantages of large transmission capacity and anti-electromagnetic interference. The potential applications range from not only broadband access, emergency/confidential communication, and cost-constrained communication occasions, but also in aerospace measurement and control, earth observation, space experiment, navigation, and positioning. Recently, fiber lasers operating around 2 μm region have become the most promising candidate in direct energy transmission and atmospheric communication because of 2 μm “eye safe” wavelength band and high transmittance up to 60% in organic gas [1].

The most conventional approaches to achieve radiation around 2 μm band are using pumped thulium-doped, holmium-doped, or thulium/holmium co-doped fibers, and related studies have been reported in the past few years [2,3,4,5]. M. Belal et al. proved that the 2 μm band optical time domain reflectometry (OTDR) system has a dynamic range of 30 dB and a spatial resolution of 10 m. The previous OTDR system works in common communication windows such as 1.3 μm, 1.5 μm, and 1.6 μm, while 2 μm band OTDR system can be adopted in the next-generation telecommunication equipment [6]. In 2013, Z. Li et al. reported the characteristics of Tm^3+^-doped fiber amplifiers in optical communication with high gain (>35 dB), low noise (5 dB), and 100 nm bandwidth at 2 μm band [7]. Z. Liu et al. reported a novel OFDM transmitter operating in 2 μm band [8].

The laser linewidth determines the modulated signal rate. The fluctuation of output optical power and spectrum leads to the accumulation of amplitude noise and the deterioration of the bit error rate at the receiving end. Thus, how to achieve high stability, narrow linewidth, and wavelength flexibility with rapid wavelength tuning or selection are the key to successful deployment for thulium-doped fibers lasers (TDFL). The current tuning devices include fiber Bragg grating (FBG) [9,10,11,12,13], F-P cavity [14,15,16], Sagnac fiber ring [17,18], Lyot filter [19,20,21], Mach–Zehnder interferometer [22,23,24], liquid crystal spatial light modulator [25], digital micromirror device (DMD) [26,27,28], and so on. Up to now, a DMD chip as a semiconductor-based addressable micromirror array has drawn considerable attention due to its flexible and fast filtering functionality in optical switching, interconnecting, and lasers. The diffracted light from selected micro-mirrors on a DMD determines its spectral filtering characteristic. In this paper, we propose a 2 μm band tunable TDFL by using a DMD chip in combination with a fixed grating. The required architecture and the characteristics are experimentally demonstrated in the next section.

## 2. Operating Principle and System Design

### 2.1. Operating Principle of TDFL

**Figure 1:**
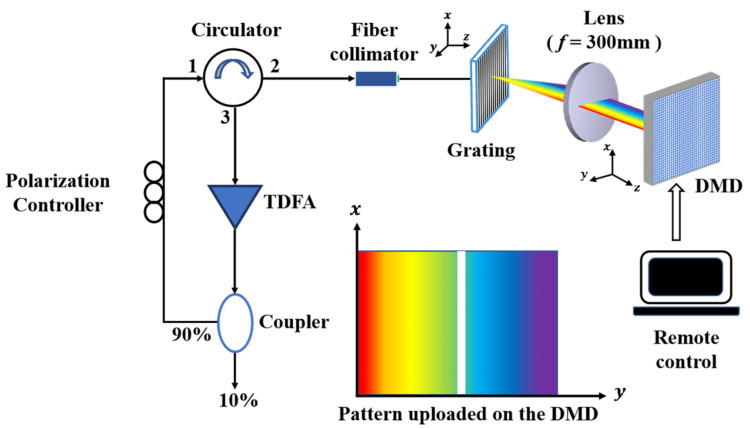
Schematic diagram of Tm-doped fiber laser based on a DMD chip. Inset is the pattern uploaded on a DMD for steering the selected beam.

Figure 1 depicts the configuration of the proposed tunable TDFL. The laser system consists of a fiber-ring resonator and an optical filter module. The fiber module includes a thulium-doped fiber amplifier (TDFA), a 90/10 coupler, a polarization controller, a fiber circulator, and a collimator. The TDFA emits the spontaneous emission spectrum (ASE) of 1910–2020 nm by adding Tm^3+^-fiber into a segment of double cladding pumped by 793 nm light. After a fiber coupler, 90% ASE light energy returns into a ring and then continues to be coupled into optical filter module via a collimator. 

A diffraction grating and DMD are placed on the front and rear focal planes of a collimating lens, respectively. The ASE spectrum from a collimator is irradiated on a grating and then produces the 1st-order dispersion spectrum in a horizontal plane. After the lens, the collimated ASE spectrum images onto different portion of a DMD. 

A DMD is an array of highly reflective aluminum micromirrors. It is an electrical input, optical output micro-electrical-mechanical system (MEMS) that allows performing high speed, efficient, and reliable spatial light modulation. During operation, the DMD controller loads each underlying memory cell with a ‘1’ or a ‘0’. Next, a mirror reset pulse is applied, which causes each micromirror to be electrostatically deflected about a hinge to the associated +/− degree state. For example, a 0.7” DMD is composed of 1024 × 768 micromirrors with a pitch 13.68 μm and fill factor 92%. Each micromirror has +/−12° tilt angle corresponding to the ‘on’ and ‘off’ states. A typical hologram is demonstrated in the inset of Figure 1. The white bar on a hologram drives the corresponding mirrors to tilt +12°, so that the corresponding waveband of ASE spectrum landing on the white area return into the collimator while the others are dropped out with dramatic attenuation, thereby realizing the laser longitudinal mode selection and wavelength tuning. The selected waveband through the collimator into a ring cavity is amplified by the TDFA, leading, after several re-circulations, to high-quality lasing generation.

### 2.2. Diffraction Performance of DMD

A DMD is adopted in TDFL as a wavelength-tunable component for selecting lasing wavelength. In experiment, we upload holograms onto a DMD by LabVIEW to control the micromirrors status. Those tilting micromirror array demonstates the diffraction effect, similar with a two-dimensional blazed grating. The diffraction performance depends on the mirror pitch, tilting angle and incident wavelength.

**Figure 2:**
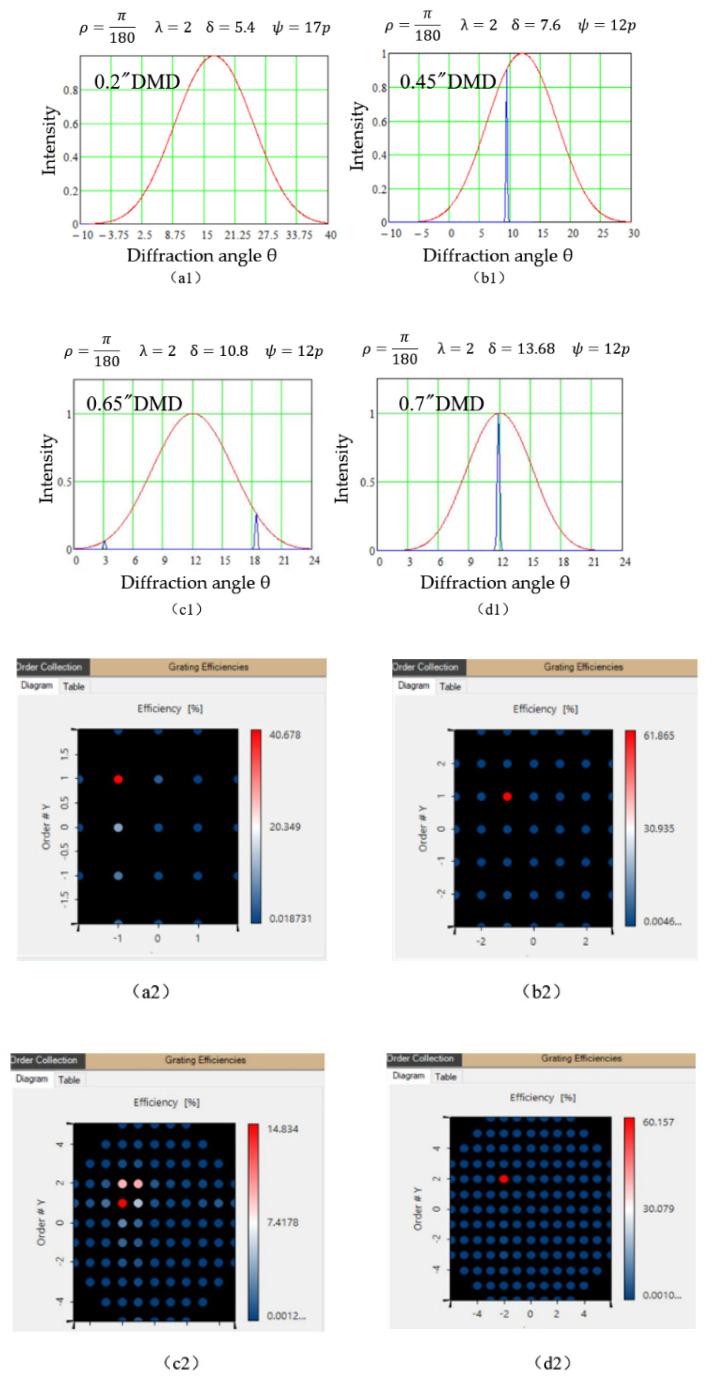
Diffraction distribution of 2 μm-wavelength light radiating on 0.2”, 0.45”, 0.65” and 0.7” DMDs at an incident angle 17° in (**a1**,**a2**) and 12° in (**b1**–**d1**,**b2**–**d2**), respectively. (**a1**–**d1**) are theoretical results on the bisecting plane (Red curves represent the single-pixel diffraction envelop and the blue curves are multi-pixel interference.). (**a2**–**d2**) are corresponding diffraction patterns in space by simulation.

In the TDFL, the configuration of a DMD must satisfy two basic conditions: (1) the diffracted light by a DMD should meet the near-blazed condition to achieve the maximum diffraction efficiency and reduce the insertion loss; (2) the selected wavelength channels must route back into the system to build a closed loop. In order to choose a suitable DMD chip operating efficiently in 2 μm band, the diffraction features of four typical DMD models launched by Texas Instruments are analyzed in detail. Table 1 shows near-infrared DMD chipsets at TI website [29]. Figure 2 demonstrates the diffraction distribution of a DMD in 2 μm band according to the two-dimensional DMD grating model that was established in our previous work [30]. 

Figure 2(a1–d1) demonstrate the diffraction distribution on the bisecting plane when 2 μm wavelength light radiates on 0.2”, 0.45”, 0.65”, and 0.7” DMDs at the incident angles 17° in Figure 2(a1,a2) and 12° in Figure 2(b1–d1,b2–d2), respectively. Based on two-dimensional DMD grating model [30], the principal maxima of multiple-pixel interference (blue peaks in Figure 2) are modulated by the single-pixel diffraction envelope (red curves). Figure 2(a2–d2) show the corresponding diffraction patterns. In Figure 2(c1,c2), when 2 μm light is incident on the 0.65” DMD, four distinct diffraction orders occur in space. Thus, the energy is not concentrated and the diffraction efficiency is lower than 14% on average, called off-blazed. In Figure 2(b1,b2,d1,d2), the diffraction energy is focused on the first order, and the diffraction efficiency is up to 60%, satisfying the ideal blazed condition. The diffracted beam from 0.45” DMD, however cannot backtrack because the corresponding diffraction angle is away from the original path, while the diffraction order from 0.7” DMD returns at 12°, exactly along the original path into the fiber ring. So the 0.7” DMD is appropriate for the closed-loop 2 μm laser.

### 2.3. Optimization of Bulk Optics 

In bulk optics, it is necessary to optimize the optical system so as to maximum utilize the DMD workspace for higher tuning accuracy. The length of ASE dispersion covering a DMD is determined by the focal length of a collimating lens. Therefore, a collimating lens with appropriate focal length is important to improve the tuning accuracy.

According to the grating equation: *d*(sin*α* + sin*β* = *kλ*), where *k* is the diffraction order (here only the first order is considered), *d* is a grating period of 459 lines/mm, *λ* is the wavelength, and *α* and *β* are the incident angle and diffraction angle, respectively. The laser operates between 1930 nm and 2000 nm and α = 17° (the blazed angle of the grating), so that *β*=36.40°@1930 nm and 38.72°@2000 nm.

We choose the focal length to be 200 mm, 300 mm, and 400 mm for comparison. The calculation shows the dispersion length on a DMD after the collimating lens are 8.1 mm, 12.1 mm, and 16.2 mm, respectively. For the size of 0.7” DMD in the horizontal axis is 14 mm, when the focal length of a collimating lens is 300 mm, the utilization ratio of the DMD is up to 86%. 

**Figure 3:**
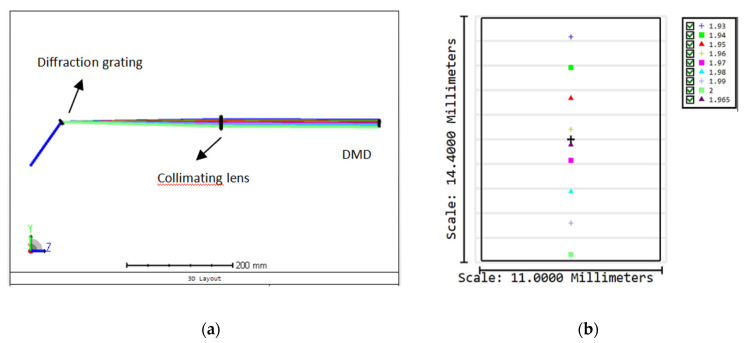
(**a**) Schematic diagram of light path in bulk optics and (**b**) dispersion spectrum of 1930–2000 nm on a 0.7” DMD.

Figure 3 shows schematic diagram of light path in bulk optics and the dispersion spectrum of 1930–2000 nm on a 0.7” DMD simulated by OpticStudio. The single pixel tuning accuracy of a DMD is defined as: $p=\delta \frac{\Delta \lambda }{L}$, where Δ*λ* = 70 nm is the tuning range of TDFL, *δ* = 13.68 μm is the pitch size, and *L* = 12.1 mm is the dispersion length. So, *p* is calculated to be 0.079 nm/pixel.

## 3. Characteristics of Tunable TDFL

**Figure 4:**
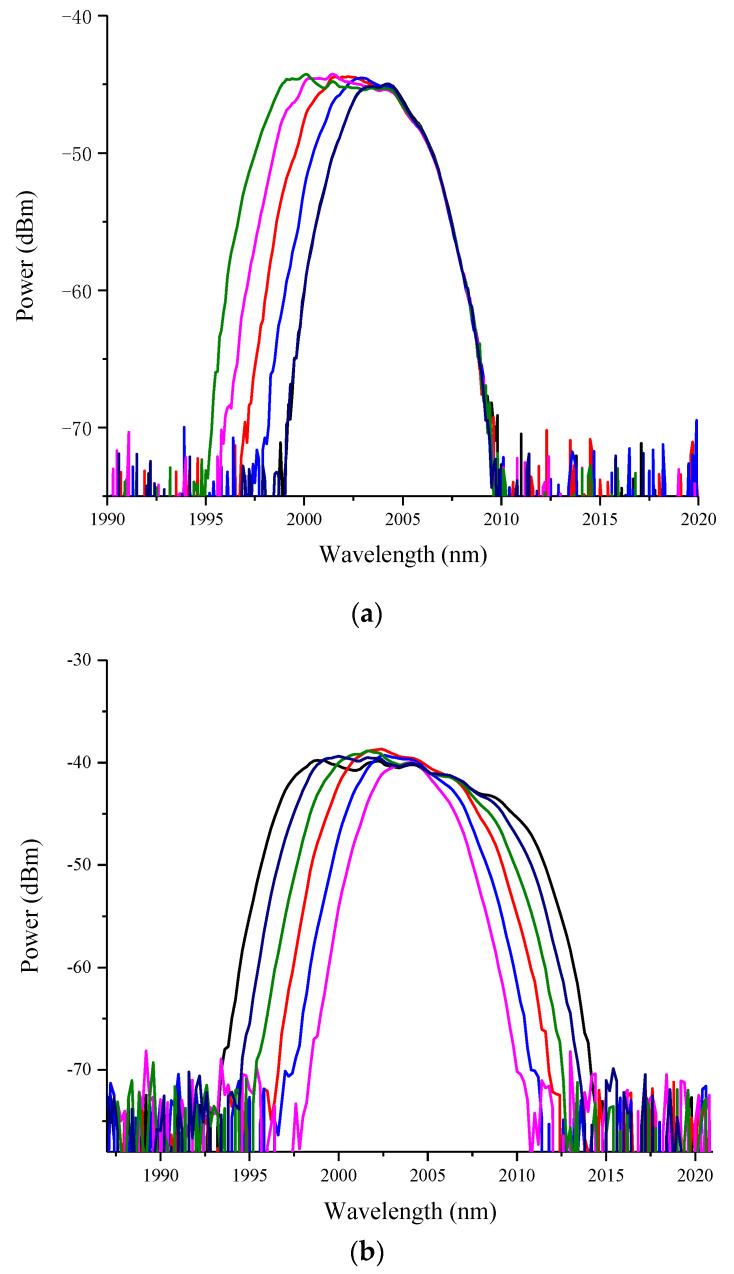
Tunable wavelength (**a**) and bandwidth (**b**) of a DMD filter.

The wavelength tunability of a DMD filter is important for the tuning performance of a laser. We upload the patterns onto the DMD to obtain signal output with different wavelength and bandwidth. Figure 4 demonstrates the tunable wavelength and flexsible bandwidth by a DMD filter in ASE spectrum of TDFA when the loop is open. The measured total loss of bulk optics from the input and output of a circulator is around 10.6 dB, which is mainly caused by the diffraction grating and DMD processor. The detailed loss of the components in the TDFL is shown in Table 2.

**Figure 5:**
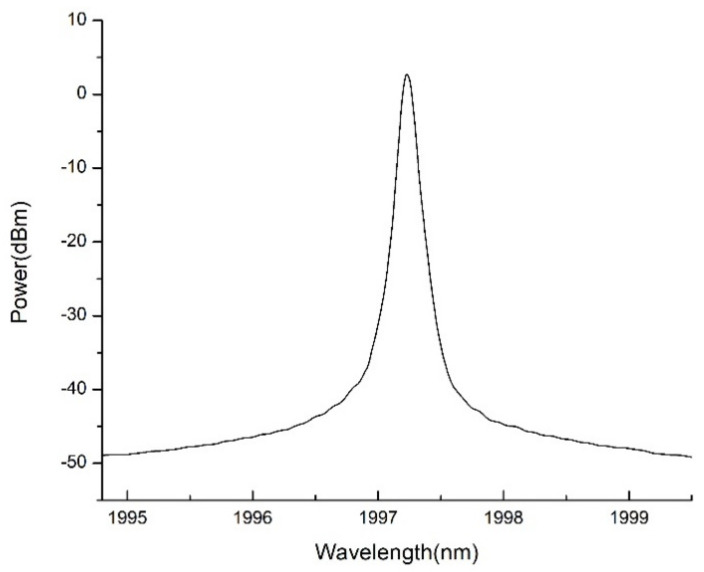
Typical output from thulium-doped fiber laser based on a DMD chip.

Figure 5 shows the typical output signal of TDFL. The laser wavelength is 1997.21 nm and the 3 dB-width is less than 0.05 nm, limited by spectrometer resolution. The side-mode suppression ratio (SMSR) is more than 50 dB.

**Figure 6:**
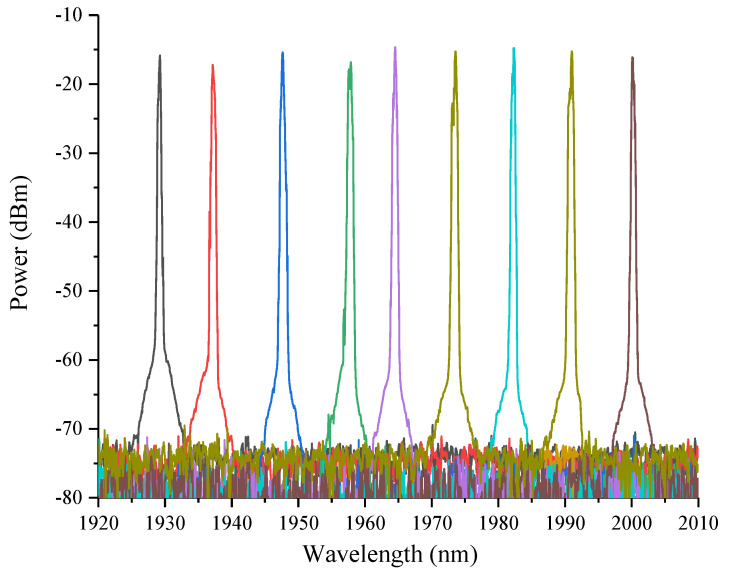
Output signal from thulium-doped fiber laser by coarse-tuning.

Figure 6 shows nine different laser spectra by coarse tuning, that exhibits high uniformity and stability. By moving the reflective area on a DMD, the wavelength tuning range from 1930–2000 nm is achieved, limited by the ASE spectrum covering a 0.7” DMD. The SMSR in whole range is greater than 50 dB. The power distribution between these wavelengths can be modified by changing the height of each reflective column on the DMD to control the wavelength dependent feedback efficiency. The tuning time of TDFL depends on the flip speed of micromirrors on a DMD chip. The switching time of this type DMD used in the system is around 80 μs.

**Figure 7:**
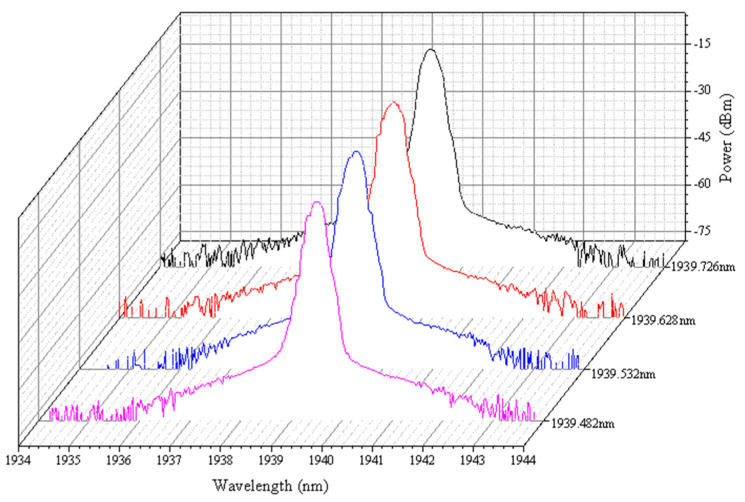
Output signals from thulium-doped fiber laser near 1939.5 nm by fine-tuning.

Figure 7 demonstrates the fine tuning output signals of TDFL around 1939.5 nm by fine tuning. The tuning accuracy is less than 0.1 nm. The reflection loss of a DMD cover glass, as well as the self-phase modulation and nonlinear effect caused by high pump power affect the laser contour [16].

**Figure 8:**
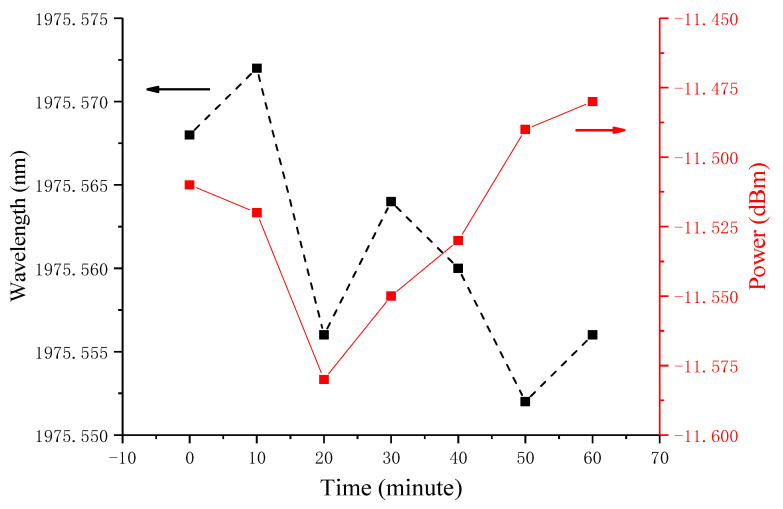
Fluctuation of output power and wavelength of thulium-doped fiber laser within 1 h.

Figure 8 shows the drift of central wavelength at 1975 nm (dotted line) and the fluctuation of peak power (solid line) when the pump power is 5 W. At room temperature, the maximum power fluctuation is 0.1 dB, and the maximum wavelength drift is less than 0.05 nm within 1 h. This fluctuation is mainly caused by the fluctuation of TDFA pumping power and the doping uniformity of the gain fiber.

## 4. Conclusions

Wavelength-tunable thulium-doped fiber laser is demonstrated based on a DMD chip. The laser achieves flexible and fast tuning of wavelength by modulating the reflective area on the DMD. The operating wavelength is continuously tunable from 1930 nm to 2000 nm with a wavelength selectivity accuracy of 0.1 nm. The 3 dB linewidth of output signals is less than 0.05 nm and the SMSR is over 50 dB. The proposed tunable TDFL has the potential applications in space communication, laser spectroscopy, medical treatment and mid-infrared systems.

## Figures and Tables

**Table 1:** Near-infrared DMD chipsets launched by Texas Instruments [29].

Type	DLP2010	DLP4500	DLP650L	DLP7000
Chip size	0.2”	0.45”	0.65”	0.7”
Micromirror array size	854 × 480	912 × 1140	1280 × 800	1024 × 768
Micromirror pitch	5.4 μm	7.6 μm	10.8 μm	13.68 μm
Micromirror tilt angle	±17°	±12°	±12°	±12°

**Table 2:** Loss from components used in TDFL in 2 μm-band.

Component	Total Loss (dB)
Circulator	1.8
Collimator	1.1
Diffraction grating	3.6
Lens	0.3
Digital Micromirror Device	3.8
Total loss	10.6

## References

[1] McComb T.S., Sims R.A., Willis C.C.C., Kadwani P., Sudesh V., Shah L., Richardson M. (2010). High-power widely tunable thulium fiber lasers. Appl. Opt..

[2] Zhang M., Kelleher E.J.R., Torrisi F., Sun Z., Hasan T., Popa D., Wang F., Ferrari A.C., Popov S.V., Taylor J.R. (2012). Tm-doped fiber laser mode-locked by graphene-polymer composite. Opt. Express.

[3] Gumenyuk R., Vartiainen I., Tuovinen H., Okhotnikov O.G. (2011). Dissipative dispersion-managed soliton 2 μm thulium/holmium fiber laser. Opt. Lett..

[4] Hemming A., Bennetts S., Simakov N., Davidson A., Haub J., Carter A. (2013). High power operation of cladding pumped holmium-doped silica fibre lasers. Opt. Express.

[5] Wang T., Ma W., Jia Q., Su Q., Liu P., Zhang P. (2018). Passively mode-locked fiber lasers based on nonlinearity at 2-µm band. IEEE J. Sel. Top. Quantum Electron..

[6] Belal M., Alam S.U., Sahu J.K., Richardson D.J., Newson T.P. Demonstration of a 2 μm-OTDR. Proceedings of the Optical Communication (ECOC 2013), 39th European Conference and Exhibition.

[7] Li Z., Heidt A.M., Daniel J.M.O., Jung Y., Alam S.U., Richardson D.J. (2013). Thulium-doped fiber amplifier for optical communications at 2 μm. Opt. Express.

[8] Liu Z., Chen Y., Wooler J.P., Kelly B., Phelan R., O’Carroll J., Wheeler N.V., Heidt A.M., Poletti F., Petrovich M.N. Up to 64QAM (30 Gbit/s) directly-modulated and directly-detected OFDM at 2 μm wavelength. Proceedings of the 2014 the European Conference on Optical Communication (ECOC).

[9] Liu S., Yan F., Peng W., Feng T., Dong Z., Chang G. (2014). Tunable Dual-Wavelength Thulium-Doped Fiber Laser by Employing a HB-FBG. IEEE Photon. Technol. Lett..

[10] He Z., Zhang P., Wu D., Wu X., He S., Wei J., Gong X., Li X., Wang T., Han K. (2020). 1.7 μm Tm-doped continue-wave and pulse fibre laser using a modulated pump based on variable pulse generated mechanisms. Opt. Laser Technol..

[11] Zhang L., Yan F., Feng T., Han W., Bai Y., Bai Z., Cheng D., Zhou H., Suo Y. (2019). Wavelength-tunable thulium-doped fiber laser with sampled fiber Bragg gratings. Opt. Laser Technol..

[12] Liu S., Yan F., Feng T., Wu B., Dong Z., Chang G.-K. (2014). Switchable and spacing-tunable dual-wavelength thulium-doped silica fiber laser based on a nonlinear amplifier loop mirror. Appl. Opt..

[13] Sabra M., Leconte B., Darwich D., Dauliat R., Tiess T., Jamier R., Humbert G., Jaeger M., Schuster K., Roy P. (2019). Widely Tunable Dual-Wavelength Fiber Laser in the 2 μm Wavelength Range. J. Light. Technol..

[14] Durán-Sánchez M., Álvarez-Tamayo R.I., Posada-Ramírez B., Ibarra-Escamilla B., Kuzin E.A., Cruz J.L., Andrés M.V. (2017). Tunable Dual-Wavelength Thulium-Doped Fiber Laser Based on FBGs and a Hi-Bi FOLM. IEEE Photon. Technol. Lett..

[15] Wang M., Huang Y., Yu L., Song Z., Liang D., Ruan S. (2018). Multi-wavelength thulium-doped fiber laser using a micro fiber-optic Fabry-Perot interferometer. IEEE Photon. J..

[16] Wang Y.P., Ju Y.L., Wu C.T., Liu W., Yang C. (2017). Wavelength-tunable thulium-doped fiber laser by employing a self-made Fabry–Perot filter. Laser Phys..

[17] Peng W., Yan F., Li Q., Liu S., Feng T., Tan S. (2013). A 1.97 μm multi-wavelength thulium-doped silica fiber laser based on a nonlinear amplifier loop mirror. Laser Phys. Lett..

[18] Chen E., Liu S., Lu P., Zhang J., Lian Z.G. (2020). Tunable 2 μm fiber laser utilizing a modified sagnac filter incorporating cascaded polarization maintaining fibers. IEEE Photon. J..

[19] Liu S., Yan F., Ting F., Zhang L., Bai Z., Han W., Zhou H. (2016). Multi-wavelength Thulium-doped fiber laser using a fiber-based Lyot filter. IEEE Photon. Technol. Lett..

[20] Sun B., Luo J., Yan Z., Liu K., Ji J., Zhang Y., Wang Q.J., Yu X. (2017). 1867–2010 nm tunable femtosecond thulium-doped all-fiber laser. Opt. Express.

[21] Ma W., Wang T., Zhang Y., Liu P., Su Y., Jia Q., Bi M., Zhang P., Jiang H. (2017). Widely tunable 2 μm continuous-wave and mode-locked fiber laser. Appl. Opt..

[22] Zhang P.Z.P., Ma W., Wang T.W.T., Jia Q.J.Q., Wan C.W.C. (2014). Stable multi-wavelength thulium-doped fiber laser based on all-fiber Mach–Zehnder interferometer. Chin. Opt. Lett..

[23] Ahmad H., Sharbirin A.S., Muhamad A., Samion M.Z., Ismail M.F.I.M.F. (2017). 2µm mode-locked thulium-doped fiber laser using Mach-Zehnder interferometer tuning capability. Laser Phys..

[24] Wei H., Zhu L., Mingli D., Fei L. (2016). A 1.8-µm multiwavelength thulium-doped fiber laser based on a hybrid interference filter. Int. J. Optomechatron..

[25] Xiao F., Alameh K., Lee T. (2009). Opto-VLSI-based tunable single-mode fiber laser. Opt. Express.

[26] Shin W., Lee Y., Yu B.-A., Noh Y.-C., Ahn T.-J. (2013). Wavelength-tunable thulium-doped single mode fiber laser based on the digitally programmable micro-mirror array. Opt. Fiber Technol..

[27] Ai Q., Chen X., Tian M., Yan B.B., Zhang Y., Song F.J., Chen G.X., Sang X.Z., Wang Y.Q., Xiao F. (2015). Demonstration of multi-wavelength tunable fiber lasers based on a digital micromirror device processor. Appl. Opt..

[28] Billaud A., Shardlow P.C., Clarkson W.A. Wavelength-Flexible Thulium-Doped Fiber Laser Employing a Digital Micro-Mirror Device Tuning Element. Proceedings of the Conference on Lasers and Electro-Optics (CLEO).

[29] Texas Instruments Near-Infrared (NIR) Chipsets—Products. https://www.ti.com/dlp-chip/advanced-light-control/near-infrared/products.html.

[30] Chen X., Yan B.-B., Song F.-J., Wang Y.-Q., Xiao F., Alameh K. (2012). Diffraction of digital micromirror device gratings and its effect on properties of tunable fiber lasers. Appl. Opt..

